# Characteristics of paediatric patients with tuberculosis and associated determinants of treatment success in Malaysia using the MyTB version 2.1 database over five years

**DOI:** 10.1186/s12889-020-10005-y

**Published:** 2020-12-10

**Authors:** S. Maria Awaluddin, Nurhuda Ismail, Yuslina Zakaria, Siti Munira Yasin, Asmah Razali, Mohd Hatta Abdul Mutalip, Noor Aliza Lodz, Kamarul Imran Musa, Faridah Kusnin, Tahir Aris

**Affiliations:** 1 Department of Public Health Medicine, Faculty of Medicine, Universiti Teknologi MARA, Sungai Buloh Campus, Jalan Hospital, 47000 Sungai Buloh, Selangor Malaysia; 2 Institute for Public Health, National Institutes of Health, Ministry of Health Malaysia, Setia Alam, Malaysia; 3 Department of Pharmaceutical Life Sciences, Faculty of Pharmacy, Universiti Teknologi MARA, Puncak Alam, Selangor Malaysia; 4 Sector of TB/Leprosy, Disease Control Division, Ministry of Health Malaysia, Putrajaya, Malaysia; 5 Department of Community Medicine, School of Medical Sciences, Universiti Sains Malaysia, Kubang Kerian, Malaysia; 6 Selangor Health State Department, Ministry of Health Malaysia, Shah Alam, Malaysia; 7 Institute for Medical Research, National Institutes of Health, Ministry of Health Malaysia, Setia Alam, Malaysia

**Keywords:** Tuberculosis, Paediatric, Children, Malaysia, Treatment success

## Abstract

**Background:**

Tuberculosis (TB) among children remains a significant public health problem in many parts of the world. The objective of this study was to describe the characteristics of TB patients and to determine the predictors of treatment success among children in Malaysia.

**Methods:**

Secondary data from MyTB version 2.1, a national database, were analysed using R version 3.6.1. Descriptive analysis and multivariable logistic regression were conducted to identify treatment success and its determinants.

**Results:**

In total, 3630 cases of TB cases were registered among children in Malaysia between 2013 and 2017. The overall treatment success rate was 87.1% in 2013 and plateaued between 90.1 and 91.4% from 2014 to 2017. TB treatment success was positively associated with being a Malaysian citizen (aOR = 3.43; 95% CI = 2.47, 4.75), being a child with BCG scars (aOR = 1.93; 95% CI = 1.39, 2.68), and being in the older age group (aOR = 1.06; 95% CI = 1.03, 1.09). Having HIV co-infection (aOR = 0.31; 95% CI = 0.16, 0.63), undergoing treatment in public hospitals (aOR = 0.38; 95% CI =0.25, 0.58), having chest X-ray findings of advanced lesion (aOR = 0.48; 95% CI = 0.33, 0.69), having EPTB (aOR = 0.58; 95% CI = 0.41, 0.82) and having sputum-positive PTB (aOR = 0.58; 95% CI = 0.43, 0.79) were negatively associated with TB treatment success among children.

**Conclusions:**

The overall success rate of treatment among children with TB in Malaysia has achieved the target of 90% since 2014 and remained plateaued until 2017. The socio-demographic characteristics of children, place of treatment, and TB disease profile were associated with the likelihood of TB treatment success among children. The treatment success rate can be increased by strengthening contact tracing activities and promoting early identification targeting the youngest children and non-Malaysian children.

## Background

Tuberculosis (TB) disease affects children aged 0 to 14 years due to recent Mycobacterium tuberculosis (mtb) infection [1, 2]. TB among children reflects the ongoing TB trasmission in the community, which needs public health attention [3]. Globally, children with TB accounted for 11% of the total TB cases in 2018 [4]. In Malaysia, children had approximately 3% of the total TB cases for the 2010–2015 Malaysian cohort [5]. In terms of TB burden, Malaysia was considered to have an intermediate TB burden with an estimated incidence rate of 92 cases per 100,000 people in 2018 [6]. Countries neighbouring Malaysia, such as the Philippines, Indonesia, Myanmar and Thailand, are among the countries with a high TB burden [4].

A study of the global estimation of TB burden among children revealed that 239,000 patients died in 2015, of whom 80% were under 5 years old, and more than 96% did not receive any prior TB treatment [7]. Data on TB among children are missing from the global surveillance database due to the non-specific clinical presentations and lack of sputum samples, which lead to underreporting and undertreatment [2]. Thus, TB among children remains a significant public health problem in many countries, including Malaysia.

TB disease is curable and preventable; therefore, the treatment success rate is an indicator of the national TB control programme (NTP) worldwide. The global success rate is set at 90% by the World Health Organization (WHO) [8]. Treatment success rates among children have ranged from 63.5 to 92.4% because hospital-based data may yield lower treatment success rates than the NTP database [9, 10]. Studies conducted among certain countries with a high TB burden reported treatment success rates above the WHO target, such as 92.4% in Pakistan and 91.7% in Iran [9, 11]. However, studies conducted in African countries indicated treatment success rates below 90% [12–14]. Treatment success in countries with a low TB burden are nearly 100% [15, 16]. Treatment success in Malaysia just achieved the WHO target of 90% but did not meet the local goal of 95% in 2020 [5].

TB treatment success among children has been associated with several determinants, such as age, sex, place of residence, citizenship, type of TB disease, HIV co-infection, nutritional status, and exposure to cigarette smoke [9, 11, 12, 14]. Danggiso et al. found that children aged 10–14 years have a higher treatment success rate and those aged 0–4 years have a lower treatment success rate than children aged 5–9 years [12]. A study conducted in Pakistan found that children who were PTB positive lived in rural residences and that younger age groups were more likely to have unsuccessful treatment [9].

TB cases among children in Malaysia differ from those reported in countries with a high TB burden in the African region, as Malaysia has a low prevalence of HIV co-infection and multidrug-resistant (MDR) TB. Furthermore, healthcare services and policies regarding TB disease in Malaysia also differ from those in neighbouring countries, such as Indonesia and the Philippines, in terms of BCG vaccination coverage and treatment cost. Therefore, utilizing surveillance data is cost-beneficial for monitoring the success of TB control programmes in general. Various studies have used secondary data to seek the determinants of treatment outcomes, focusing either on treatment success or on unsuccessful treatment [9, 12, 14]. The first objective of the current study was to describe the characteristics of TB patients and to determine the rate of treatment success according to the socio-demographic profiles of children in Malaysia. The second objective was to determine the predictors of treatment success among children with TB in Malaysia.

## Methods

### Data and sampling

The study utilized a cross-sectional study design to analyse secondary data from MyTB version 2.1, a national TB surveillance database. It has used an online data entry system since 2011 and contains all TB case registrations for each year. The online system has enabled designated health staff in each district of the 13 states and three federal territories in Malaysia to register TB cases and related information, including socio-demographic profiles, TB investigations, TB diagnosis, medication and treatment outcomes. The data were monitored by the TB sector at the state level and finally at the national level. The MyTB version 2.1 manual adopted the previous version of the Tuberculosis Information System (TBIS), which used hardcopy documentation. Standard definitions were used for each variable in MyTB version 2.1. Certain variables still had higher rates of missing data, such as household income, education level and the number of household dependents. There were no variables to assess the socio-economic status of the parents of children with TB in this database. For this study, the analysis was conducted with data from 2013 to 2017. An optimal sample size of 2911 cases was needed to obtain statistically meaningful findings. The total number of registered TB cases among children was 3630 for 2013–2017.

### Variables definition

Treatment outcome was defined according to the WHO guidelines, “Definitions and reporting framework for tuberculosis–2013 revision”. Treatment outcomes consisted of six categories: cured, completed, failed, death, lost to follow-up and not evaluated. These categories can further be classified into two groups, treatment success and unsuccessful treatment. A child who has completed treatment and shows evidence of being cured as reflected in sputum status is considered cured. A child who has completed treatment but has no confirmed sputum result is categorized as completed because he/she might have sputum-negative PTB or EPTB. The unsuccessful treatment group comprised children who failed treatment, those who died, those lost to follow-up and those who were not evaluated [17].

The independent variables were socio-demographic variables such as age, sex, citizenship and ethnicity. Malaysia is a multi-ethnic country, with the major ethnicities being Malay, Chinese and Indian. Other ethnicities included Bumiputera Sabah and Bumiputera Sarawak. In this study, “Others” refer to Malaysian citizens whose ethnicities are not mentioned above. Rural and urban residents were categorized based on the definition from the Department of Statistics Malaysia. Localities were classified as urban areas if they were under city council management and had a population of 10,000 and above [18]. TB disease profiles included variables such as TB disease diagnosis, sputum result, chest X-ray findings and HIV co-infection status.

### Data analysis

Data were analysed using R for Windows, Version 3.6.1 [19]. The Summary Tools package [20] was used to conduct a descriptive analysis by tabulating the frequency table and contingency table with the chi-square test results. The generalized linear model (GLM) family logit function under the CAR package [21] was used to measure the odds ratios in simple and multivariable logistic regression tests. For model fit assessment, the Resource Selection package [22] was utilized to calculate the Hosmer-Lemeshow goodness of fit. Simple logistic regression was conducted to assess the association between the individual independent variables and the dependent variable. All the independent variables were included in the final model based on a *p*-value less than 0.25 [23], based on clinically relevant and supporting evidence from the literature review. The preliminary main effect model included eleven independent variables in the analysis, and two independent variables were excluded from the final main effect model due to significant interaction terms. Model fit and regression diagnostics were assessed to ensure a robust model. Finally, the MICE package was used to perform a multiple imputation model [24] because the main effect model was analysed only for 3132 out of 3550 cases. Multiple imputations via the chained equation method were conducted on a variable-by-variable basis for each incomplete pair of tested variables. The results for the adjusted odds ratios (aORs) were reported based on the multiple imputation model.

## Results

This study analysed data from a total of 3630 registered TB cases among children in Malaysia from 2013 to 2017. However, the final analysis included only 3550 cases because the other cases had missing data on treatment outcomes or because the final diagnosis changed to something other than TB. Table 1 illustrates the trends of TB case registration and treatment success among children from 2013 to 2017. The overall rate of treatment success among children with TB in Malaysia was 90.1%.

**Table 1:** Data on TB cases registration in MyTB version 2.1 and TB treatment success among children in Malaysia, 2013–2017

Years	Number of registered cases in MyTB version 2.1					The proportion of paediatric cases (%)	Treatment outcome according to age groups (%), *n* = 3550			
	All cases	0–4 years	5–9 years	10–14 years	0–14 years		0–4 years	5–9 years	10–14 years	0–14 years
2013	24,069	279	144	314	737	3.1	79.0	93.0	91.2	87.1
2014	24,701	244	141	315	700	2.8	87.6	89.9	92.9	90.4
2015	24,160	275	142	328	745	3.1	88.5	92.9	93.2	91.4
2016	25,736	255	144	311	710	2.8	88.7	92.0	93.1	91.3
2017	26,159	300	136	302	738	2.8	87.1	92.5	91.9	90.1

Table 2 shows the results of descriptive analysis of the registered TB cases among children in Malaysia. In terms of ethnicity, 42.5% of the TB patients among children in Malaysia were Malays, followed by 19.8% Bumiputera Sabah, 10.7% Bumiputera Sarawak, 13.5% Non-Malaysian, 5.4% Chinese, 4.4% Others, and 3.6% Indian. The highest proportion of TB cases was among children aged 10–14 years, followed by children aged 0–4 years and those aged 5–9 years (43.5, 37.0 and 19.5%, respectively). Of the 479 TB cases among non-Malaysian children, the highest proportion was from the Philippines, followed by Indonesia and Myanmar (55.5, 24.6 and 16.1%, respectively). A total of 94.3% (2897/3701) of Malaysian citizens had BCG scars, whereas only 36.5% (175/479) of non-Malaysian citizens had BCG scars.

**Table 2:** Descriptive analysis of children with TB in Malaysia, 2013–2017

Parameters	n	(%)
Socio-demographic profiles		
Age (median, IQR) (n: 3317)	8.5 (10.5)	–
Age groups		
0–4	1313	37.0
5–9	691	19.5
10–14	1546	43.5
Sex		
Male	1726	48.6
Female	1824	51.4
Citizenship		
Malaysian	3071	86.5
Non-Malaysian	479	13.5
Ethnicity (n: 3546)		
Malay	1508	42.5
Chinese	192	5.4
Indian	127	3.6
Bumiputera Sabah	703	19.8
Bumiputera Sarawak	381	10.7
Others (Malaysian)	156	4.4
Non-Malaysian	479	13.5
Place of residence (n: 3516)		
Urban	1801	51.2
Rural	1715	48.8
Health utilization		
Treatment centre one		
Public hospital	2802	78.9
Public clinic	579	16.3
Private facilities	169	4.8
TB disease profiles		
Category of cases		
New case	3461	97.5
Re-treatment	89	2.5
Method of detection		
Passive	2827	79.6
Active	723	20.4
BCG scar		
Yes	3072	86.5
No	478	13.5
CXR finding (n: 3380)		
No lesion	895	26.5
Minimal	1741	51.5
Advanced	744	22.0
HIV co-infection		
Yes	48	1.4
No	3502	98.6
TB disease profiles		
TB disease diagnosis		
^a^PTB sputum -ve	1606	45.2
PTB sputum +ve	976	27.5
EPTB	968	27.3
TB meningitis		
Yes	153	4.3
No	3397	95.7
TB miliary		
Yes	67	1.9
No	3483	98.1
Treatment outcomes		
Cured	844	23.8
Completed	2353	66.3
Death	127	3.6
Failed treatment	4	0.1
Lost to follow up	65	1.8
Not evaluated	157	4.4

^a^The PTB sputum -ve group, included PTB sputum not done (89 cases)

Table 3 shows the rates of TB treatment success according to the socio-demographic and TB disease profiles. The percentage of TB treatment success was significantly higher among older children and children who were Malaysian citizens. Having TB detected through active findings, having chest X-ray findings of no or minimal lesion, not having human immunodeficiency virus (HIV) co-infection and having sputum-negative PTB were also associated with higher treatment success rates. Children who underwent treatment in a public clinic or private facility had higher treatment success than those treated in a public hospital.

**Table 3:** Treatment outcomes among children with TB in Malaysia, 2013–2017

Parameters	^a^Successful		^b^Unsuccessful		*P-value*
	n	%	n	%	
Overall	3197	90.1	353	9.9	–
Socio-demographic profiles					
Age (median, IQR)	8.9 (10.6)		5.0 (11.1)		< 0.001
Age groups (years)					
0–4	1131	86.1	182	13.9	
5–9	636	92.0	55	8.0	
10–14	1430	92.5	116	7.5	< 0.001
Sex					
Male	1538	89.1	188	10.9	
Female	1659	91.0	165	9.0	0.075
Citizenship					
Malaysian	2857	93.0	214	7.0	
Non-Malaysian	340	71.0	139	29.0	< 0.001
Place of residence					
Urban	1632	90.6	169	9.4	
Rural	1534	89.4	181	10.6	0.270
Health utilization					
Treatment centre one					
Public hospital	2489	88.8	313	11.2	
Public clinic	549	94.8	30	5.2	
Private facilities	159	94.1	10	5.9	< 0.001
TB disease profile					
Method of detection					
Passive	2524	89.3	303	10.7	
Active	673	93.1	50	6.9	0.003
BCG scar					
Yes	2850	92.8	222	7.2	
No	347	72.6	131	27.4	< 0.001
CXR finding					
No lesion	819	91.5	76	8.5	
Minimal	1607	92.3	134	7.7	
Advanced	620	83.3	124	16.7	< 0.001
HIV co-infection					
Yes	35	72.9	13	27.1	
No	3162	90.3	340	9.7	< 0.001
TB diagnosis					
PTB SS -ve	1473	91.7	133	8.3	
PTB SS + ve	859	88.0	117	12.0	
EPTB	865	89.4	103	10.6	0.007

^a^successful: the group of cured and completed

^b^unsuccessful: the group of failed, died, lost to follow up, not evaluated

Table 4 shows the results of simple logistic regression and multivariable logistic regression to determine the predictors of TB treatment success among children with TB in Malaysia. TB treatment success was positively associated with being a Malaysian citizen (aOR = 3.43; 95% CI = 2.47, 4.75), have BCG scars (aOR = 1.93; 95% CI = 1.39, 2.68), and being in the older age group (aOR = 1.06; 95% CI = 1.03, 1.09. Having HIV co-infection (aOR = 0.31; 95% CI = 0.16, 0.63), receiving treatment in public hospitals (aOR = 0.38; 95% CI =0.25, 0.58) having chest X-ray findings of advanced lesion (aOR = 0.48; 95% CI = 0.33, 0.69), having EPTB (aOR = 0.58; 95% CI = 0.41, 0.82) and having sputum-positive PTB (aOR = 0.58; 95% CI = 0.43, 0.79) were negatively associated with TB treatment success among children.

**Table 4:** Multivariable logistic regression for the determinants of treatment success among children with TB in Malaysia, 2013–2017

Parameters	Crude OR	95% CI		Model 1 aOR	95% CI		*P- value*	Model 2 ^a^aOR (MICE)	95% CI		*P-value*
		Lower	Upper		Lower	Upper			Lower	Upper	
Socio-demographic profiles											
Age	1.06	1.04	1.09	1.05	1.03	1.08	< 0.001	1.06	1.03	1.09	< 0.001
Sex											
Male	ref	–	–	–	–	–	–	–	–	–	–
Female	1.23	0.99	1.53	1.00	0.77	1.30	0.987	1.11	0.88	1.41	0.384
Citizenship											
Malaysian	5.39	4.23	6.85	4.21	2.89	6.11	< 0.001	3.43	2.47	4.75	< 0.001
Non-Malaysian	ref	–	–	ref	–	–	–	ref	–	–	–
Place of residence											
Urban	1.14	0.91	1.42	0.91	0.70	1.18	0.485	0.93	0.73	1.19	0.563
Rural	ref	–	–	–	–	–	–	–	–	–	–
Treatment centre one											
Public hospital	0.43	0.29	0.63	0.37	0.21	0.61	< 0.001	0.38	0.25	0.58	< 0.001
Public clinic	ref	–	–	–	–	–	–	ref	–	–	–
Private facilities	0.87	0.43	1.91	0.78	0.34	1.94	0.581	0.79	0.36	1.71	0.544
TB disease profile											
BCG scar											
Yes	4.85	3.8	6.17	1.88	1.28	2.72	0.00102	1.93	1.39	2.68	< 0.001
No	ref	–	–	ref	–	–		ref	–	–	
CXR finding											
No lesion	ref	–	–	ref	–	–		ref	–	–	
Minimal	1.11	0.83	1.49	0.97	0.66	1.41	0.868	1.01	0.70	1.44	0.976
Advanced	0.46	0.34	0.63	0.48	0.32	0.71	< 0.001	0.48	0.33	0.69	< 0.001
HIV co-infection											
Yes	0.29	0.16	0.57	0.27	0.13	0.58	< 0.001	0.31	0.16	0.63	0.001
No	ref	–	–	ref	–	–		ref	–	–	
Type of TB											
PTB SS -ve	ref	–	–	ref	–	–		ref	–	–	
PTB SS + ve	0.66	0.51	0.86	0.62	0.44	0.86	0.004	0.58	0.43	0.79	< 0.001
EPTB	0.76	0.58	0.99	0.55	0.38	0.80	0.002	0.58	0.41	0.82	0.002

Summary of model fit for Model 1:

Hosmer-Lemeshow goodness of fit: *p*-value = 0.1108

Likelihood ratio test: *p*-value < 0.001

Classification table: 90.6%

Area under the curve: 0.73 (95% CI: 0.70–0.77)

Akaike information criterion: 1729.1

^a^aOR MICE: Adjusted odds ratio with multivariate imputation by chained equations

## Discussion

This study describes the characteristics of children with TB in Malaysia from 2013 to 2017 using the recommended NTP surveillance database. TB incidence among children is monitored according to age group, as younger children constitute a high-risk group for TB disease complications [25]. The standard age disaggregation for WHO TB reporting among children is as follows: 0–4 years and 5–14 years [2]. Other researchers have divided age groups into three categories: 0–4 years, 5–9 years, and 10–14 years [12, 14]. A few studies in countries with a low TB burden have used different cut-off points to define paediatric TB, such as being under 18 years [16, 26] or 0–16 years [15]; in such cases, inter-country comparison was unsuitable.

The proportion of registered TB cases among children in Malaysia was less than 5% compared to the total registered cases. The ratio of TB cases was much lower among the age group 0–4 years than among the older group. Regarding these findings, the national database is still far from the WHO benchmarks for a higher-quality database for children with TB disease [27]. The most cases were in children aged 10–14 years, followed by those aged 0–4 years. In particular, the youngest age group has a 30 to 40% risk of developing PTB disease and a 10 to 20% of developing TB meningitis or miliary TB following Mtb infection. The risk of PTB disease was 2% for children aged 5–9 years and between 10 and 20% for those aged 10–14 years [28]. Younger children have immature immune systems, while adolescents have greater social contact, leading to a greater risk of TB infection [29]. The number of registered TB cases was low for children aged 0–4 years because there were more missing cases, even though Malaysia does not have a high TB burden. A known challenge for cases among children is accurately diagnosing TB because of the non-specific symptoms and paucibacillary. A study conducted among countries with a high TB burden found a low number of cases among the 0–4-year age group compared to the older age group, which indicates the possibility of missing cases among the younger age groups [11, 12, 30].

The proportion of registered TB cases among children in Malaysia can be broken down by the main ethnicities in Malaysia, including non-Malaysian citizens, who accounted for 13.5% of cases. Among immigrants, a higher proportion of TB patients originated from neighbouring countries such as the Philippines, Indonesia and Myanmar, countries with a high TB burden [4]. Similar observations have been made among registered TB cases in countries with a low TB burden, such as Israel, Australia and Canada, where a substantial proportion of TB cases were immigrants from countries with a high TB burden [15, 16, 31].

The overall rate of treatment success among children in Malaysia was 90.1%, which achieved the WHO target of 90%, except for children under 5 years old. The Malaysian goal of a 95% treatment success rate in 2020 seems unachievable [5]. The rates of treatment success in other countries varied because of the wide range of data source used, either from an NTP database or review of hospital-based records. Hospital-based data [30, 32] usually reflect a small number of TB cases compared to those from the NTP database [14]. Moreover, hospital-based studies often showed a higher rate of severe TB disease [33] and thus a lower proportion of treatment success. Two kinds of databases are useful to monitor the outcomes of TB, whether success or unsuccessful treatment [9, 33, 34]. The MyTB version 2.1 database includes a large number of variables, such as place of treatment, BCG scars, treatment regime, directly observed therapy (DOT) for the intensive phase, and DOT supervisor. However, this database needs improvement regarding the documentation of household income because there are many missing values. Nevertheless, nine independent variables were selected as determinants of treatment success among children in Malaysia.

The age of children independently determines their likelihood of treatment success when the analysis includes a continuous variable. The findings differed from previous studies in which the analysis employed a categorical variable [9, 14]. However, the conclusions of this study and other studies were similar in that the older age group had a higher likelihood of treatment success than the 0–4-year age group [14, 35]. In this study, the categorical variable of age exhibited a significant interaction with other determinants, such as TB disease diagnosis and BCG scars, which led to biased estimates.

In terms of citizenship as a determinant of treatment success, Malaysian citizens had a higher likelihood of treatment success than non-Malaysians. This finding was in line with the national TB policy stating that TB treatment is free of charge. TB treatment in Malaysia utilizes a holistic approach including case detection, a DOT programme and TB counselling, effective follow-up and a BCG vaccination policy for newborns [5]. On the other hand, the non-Malaysian group included children from countries with a high TB burden and low BCG coverage. Although TB treatment is free in Malaysia, the initial evaluation procedure before the confirmation of TB disease is expensive. The associated financial burden may prevent non-Malaysian families from visiting the clinic at an early stage of health changes.

TB was diagnosed in children in Malaysia primarily by sputum-negative PTB, followed by sputum-positive PTB and EPTB. The pattern of TB diagnosis among children was similar to that in previous studies [9, 14]. Studies from hospital-based settings report a higher percentage of EPTB than PTB and a higher number of more severe TB types, such as TB meningitis [33, 36]. Younger age groups have difficulty producing sputum samples; hence, sputum examination results rarely become positive for younger groups compared with older age groups [37]. Children with sputum-positive PTB and EPTB have a low likelihood of treatment success; in fact, children in EPTB groups may present with TB meningitis, a severe type of TB disease [33]. Children with sputum-positive PTB presented with a higher load of Mtb in their bodies, which triggered an excessive immune reaction that contributed to the probability of unsuccessful treatment among adolescents [9, 14, 29]. In addition, chest X-ray findings were associated with treatment success; children with chest X-ray results of advanced lesion were observed to have a low success rate compared with the rate for children whose chest X-ray showed no lesion or minimal lesion. Previous studies conducted in Ethiopia and Pakistan excluded chest X-ray results due to the availability of variables in their NTP database [9, 12, 14]. Recent local studies found an association of chest X-ray findings with unfavourable outcomes; however, these studies were conducted among all age groups [38, 39].

TB disease is associated with HIV co-infection because HIV alters the human immune system. HIV co-infection accounted for only 1.4% of the total TB cases among children in this study. Malaysia still has a low occurrence of HIV co-infection, but those who had HIV co-infection had low odds for TB treatment success. This finding was similar to the observations in other countries with a higher prevalence of HIV co-infection [14, 30, 35].

BCG vaccination provides protection from TB meningitis and miliary TB among the age group 0–4 years and some protection from PTB until the age of 14 years [40]. Considering this fact, 86.5% of our registered TB cases among children had BCG scars. Children without BCG scars were more likely to be non-Malaysian citizens (304/479, 63.5%) than Malaysian citizens (174/3071, 5.7%). Children with BCG scars had a higher likelihood of treatment success than those without BCG scars in this study, possibly because they have been protected from severe TB. However, previous research in China did not find a significant association between BCG scars and TB treatment success with the utilization of hospital databases [33]. Many other studies in Ethiopia and Pakistan did not include the presence of BCG scars as a determinant of treatment success due to data availability in their NTP system [9, 12, 14, 35].

Place of treatment was another predictor of TB treatment success Children who were treated at a public hospital were less likely to succeed than those treated in a public clinic. Thus, cases in the hospital were more severe and difficult to manage. A local study using a Malaysian cohort in 2012 observed that a patient treated in a public hospital or clinic had a higher likelihood of unfavourable outcomes than those treated in a private facility; however, that study included all age groups [38]. Studies conducted in countries with a high TB burden did not include the place of treatment in their model; hence, comparisons cannot be made. Health facilities providing TB treatment should ensure that their services are patient-friendly to promote treatment success. In addition, early detection during active case evaluation, especially among younger age groups, may reduce the number of severe TB cases that require hospital management.

The place of residence for children showed a non-significant result in the final model. Thus, the country’s health services did not differ in terms of the accessibility and quality of TB care between urban and rural areas. A study conducted in Pakistan, with a high TB burden, observed that children from a rural area had higher odds of unsuccessful treatment [9], possibly because children in urban areas have greater access to health facilities, are better educated and are more confident taking modern medicine.

Among the socio-demographic determinants of treatment success or unsuccessful treatment outcomes, no association with children’s sex was found in previous studies [9, 11]. Similar findings were reported in our research, concluding that TB treatment success among children did not differ by sex.

### Limitations and strength of the study

The study used secondary data that depended upon the availability of relevant parameters. A few variables could not be included because of the large amount of missing data and lack of valid definitions, such as those for household income, number of household dependents and education level. Thus, many predictors, such as economic status, parental education level and BMI status of the child, cannot be assessed in this study. Second, this study used a cross-sectional design; therefore, a causal relationship between the dependent and independent variables could not be established. The main strength of this study was the utilization of 5 years of data on TB case registration among children in Malaysia. The sample size in this research was adequate to generate statistically meaningful findings. Although secondary data analysis might have led to more missing values, only 1.6% of the values in the selected dataset were missing. The use of secondary data depends upon the percentage of missing data and the standard definition in the manual.

## Conclusions

The overall rate of treatment success among children with TB in Malaysia has achieved the target of 90%, except for children under 5 years of age. Treatment success among children with TB in Malaysia is positively associated with being a Malaysian citizen, being an older child and having BCG scars. Having HIV co-infection, being treated in public hospitals, having chest X-ray findings of advanced lesion, having sputum-positive PTB, and having EPTB were negatively associated with TB treatment success. Strategies for improving rates of treatment success include strengthening contact tracing activities and early identification among specific groups, such as the youngest and non-Malaysian groups.

## Data Availability

The dataset of MyTB version 2.1 for 2013–2017 is available; anyone requesting this dataset should consult the TB Sector in the Ministry of Health, Malaysia.

## Abbreviations

aOR: Adjusted odds ratios
BCG: Bacillus Calmette–Guérin
CAR: Companion to applied regression
CXR: Chest X-ray
DOT: Directly observed treatment
EPTB: Extrapulmonary tuberculosis
GLM: Generalized linear model
HIV: Human immunodeficiency virus
IQR: Interquartile range
MDR: Multidrug-resistant
MICE: Multivariate imputation by chained equations
Mtb: *Mycobacterium tuberculosis*
MyTB: Malaysian Tuberculosis Database
NTP: National Tuberculosis Control Programme
PTB: Pulmonary tuberculosis
TB: Tuberculosis
TBIS: Tuberculosis Information System
WHO: World Health Organization
